# Posterior laryngeal web in an adult with GERD-associated globus pharyngeus: A rare case managed conservatively

**DOI:** 10.1093/omcr/omaf231

**Published:** 2025-11-26

**Authors:** Samiksha Lamichhane, Asitama Sarkar, Silvia Maharjan, Roshan Shrestha, Shritik Devkota, Prajjwal Pokhrel, Harsimran Bhatia

**Affiliations:** Department of Radiodiagnosis and Imaging, BP Koirala Institute of Health Sciences, Dharan, 56700, Nepal; Department of Otorhinolaryngology and Head and Neck Surgery, Anil Baghi Hospital, Punjab, 152002, India; Health Foundation Nepal, Ghorahi, Dang, 45100, Nepal; Department of Emergency Medicine, Ratnanagar, Chitwan, 44204, Nepal; Department of Radiodiagnosis and Imaging, Anil Baghi Hospital, Punjab, 152002, India; Health Foundation Nepal, Ghorahi, Dang, 45100, Nepal; Department of Radiodiagnosis and Imaging, Post Graduate Institute of Medical Education and Research, Chandigarh, 160012, India

**Keywords:** laryngeal web, gastroesophageal reflux disease (GERD), airway obstruction, globus Pharyngeus, anti-reflux therapy

## Abstract

Laryngeal webs are rare structural anomalies, typically anterior in location when congenital. Posterior laryngeal webs are exceedingly uncommon and are most often acquired, with common associations including prior intubation and gastroesophageal reflux disease (GERD). GERD-induced laryngeal inflammation is a proposed mechanism for web formation due to chronic mucosal irritation and fibrosis. We present the case of a 24-year-old male with recurrent globus pharyngeus and chest discomfort, found to have a posterior laryngeal web in the absence of prior intubation or trauma. The patient was diagnosed with GERD and demonstrated marked symptomatic improvement following anti-reflux therapy. This case highlights the potential link between GERD and posterior laryngeal web formation, emphasizing the role of conservative management in select cases. Further research is warranted to explore the pathophysiological relationship between GERD and laryngeal structural changes, as well as to refine treatment strategies.

## Introduction

Laryngeal webs are rare anomalies resulting from abnormal embryonic development of the larynx and are typically congenital in origin [1]. Congenital laryngeal webs are almost always located anteriorly, whereas posterior laryngeal webs are generally acquired and associated with various etiological factors. Among these, prolonged or traumatic intubation; especially involving the posterior glottis is considered the most common cause in adults [2]. Gastroesophageal reflux disease (GERD) has also been proposed as a significant contributing factor [1]. In many cases, however, no clear etiology is identified, and such instances are termed ‘idiopathic,’ with sporadic reports in the literature [1, 2]. A multidisciplinary approach is often essential for accurate diagnosis and effective management. While surgery remains the mainstay of treatment for laryngeal webs, anti-reflux therapy has emerged as an important adjunct in cases where laryngopharyngeal reflux is implicated [1–4].

In this report, we present the case of a 24-year-old male who visited the ENT outpatient department with complaints of recurrent globus pharyngeus and had no history of prior intubation. On subsequent evaluation, a posterior laryngeal web was incidentally identified.

## Case report

A 24-year-old male presented with a one-year history of recurrent throat discomfort described as a persistent sensation of a foreign body, along with intermittent belching and mild chest discomfort. He was a non-smoker, consumed alcohol occasionally, and had no significant medical or surgical history. There were no symptoms of dyspnea, stridor, or voice changes.

**Figure 1:**
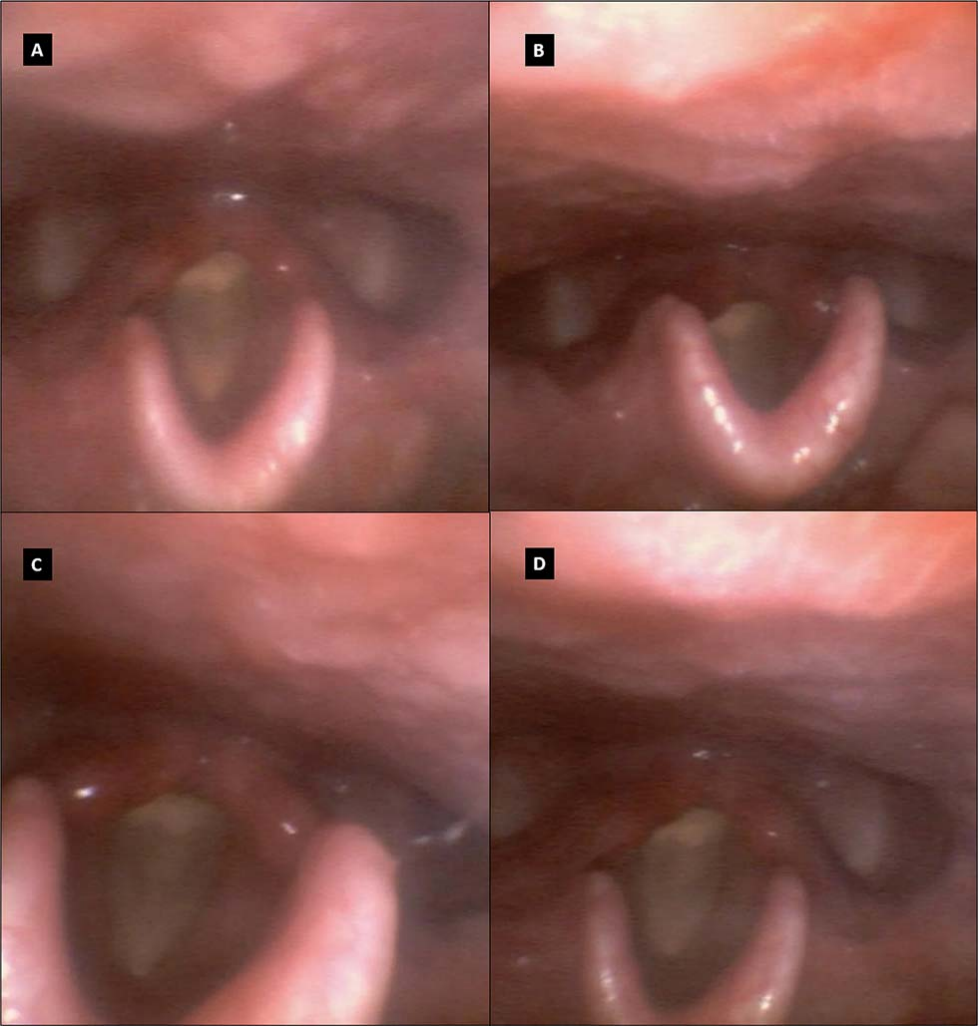
Flexible videolaryngoscopy image showing the glottic web when vocal folds are in adduction (A, B) and abduction (C, D).

Physical examination was unremarkable except for a deviated nasal septum. Throat and nasal mucosa appeared normal. Flexible video laryngoscopy (fig. 1) revealed a thin, white membranous structure covering most of the glottic aperture, along with interarytenoid pachyderma. Repeated coughing did not dislodge the membrane, suggesting a posterior laryngeal web.

**Figure 2:**
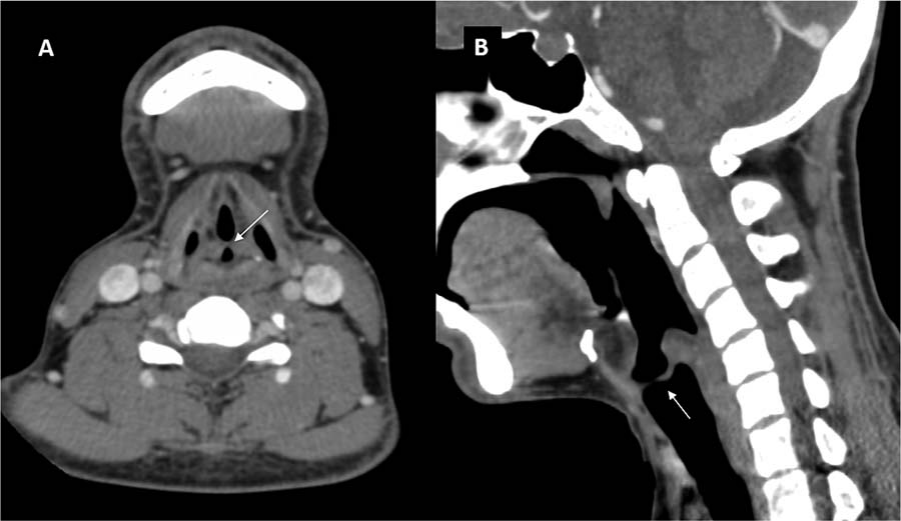
CECT axial (A) and sagittal (B) images showing a shelf like soft tissue structure (arrows) along posterior aspect of the glottic with minimal narrowing of glottic airway.

A CT scan of the neck confirmed a linear, shelf-like soft tissue structure (1.7 mm thick) extending from the posterior glottis with minimal airway narrowing (fig. 2).

The patient was referred to gastroenterology. Upper GI endoscopy revealed a small hiatal hernia and mild esophagitis. *Helicobacter pylori* infection was confirmed by a positive rapid urease test. A 24-hour pH monitoring study confirmed gastroesophageal reflux disease (GERD).

He was started on bismuth-based quadruple therapy and transitioned to long-term lansoprazole. At three months, the patient reported significant symptom improvement and opted against follow-up laryngoscopy due to symptom resolution.

## Discussion

This case report presents a curious case of an adult patient with an incidentally detected posterior laryngeal web in a patient with LPRD and GERD. Laryngeal webs are uncommon congenital lesions that can cause airway obstruction, but they are typically located anteriorly [1, 2]. The presence of a posterior laryngeal web in an adult patient with GERD is an unusual finding. Table 1 provides brief differentiation of anterior and posterior laryngeal webs.

**Table 1:** Difference between anterior and posterior laryngeal web.

Feature	Anterior Laryngeal Web	Posterior Laryngeal Web
Etiology	Mostly congenital	Rare; usually acquired, often post-intubation, trauma, or chronic inflammation (e.g. LPRD/GERD)
Location	Anterior glottis, near vocal folds	Posterior glottis, interarytenoid region
Typical Symptoms	Respiratory distress, stridor, weak or high-pitched cry (in infants), voice changes	Chronic cough, globus sensation, occasional voice changes; often asymptomatic
Diagnosis	laryngoscopy	Flexible laryngoscopy supported with clinical history
Management	Usually surgical (excision, laser)	Conservative if asymptomatic; surgery if symptomatic (airway or voice issues)

Posterior laryngeal webs are most commonly associated with prior endotracheal intubation. Other reported etiologies include laryngeal trauma, infectious diseases such as diphtheria and tuberculosis, ingestion of foreign bodies, smoke inhalation, and radiotherapy [2–4].

**Figure 3:**
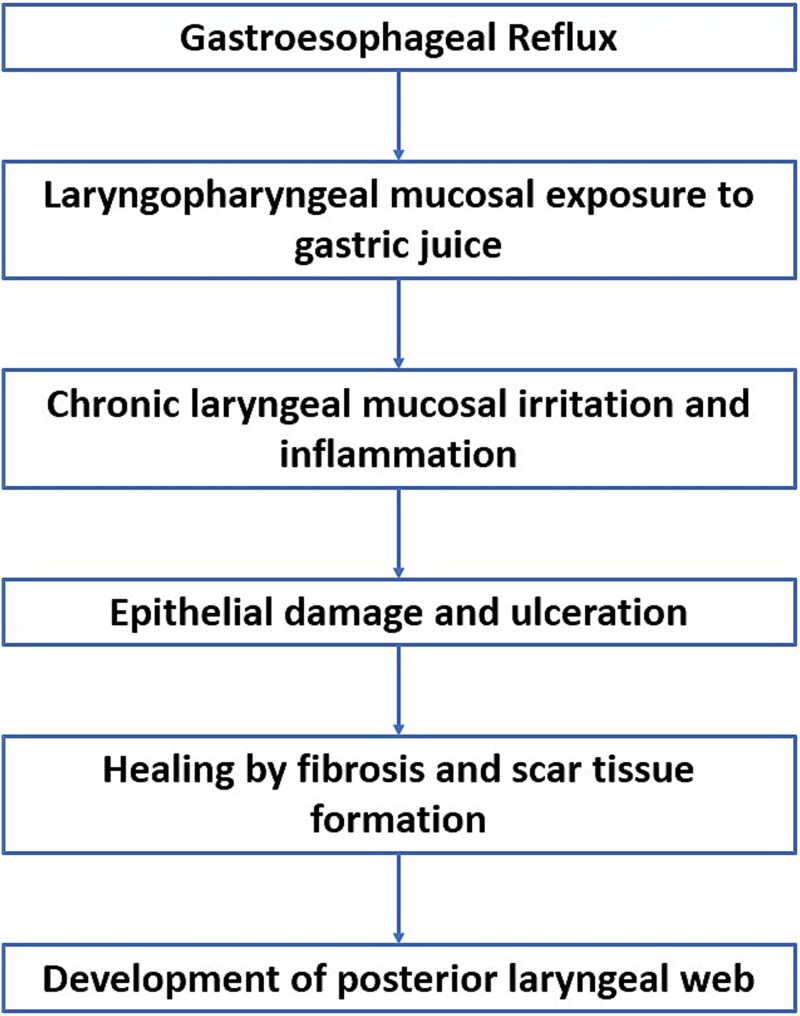
Possible pathophysiological mechanism linking GERD to posterior laryngeal web formation.

GERD is a well-established cause of globus pharyngeus, a persistent sensation of a lump in the throat [5]. Studies indicate that reflux can lead to inflammation and injury of the laryngeal mucosa, resulting the conditions such as laryngeal mucosa, resulting in conditions such as laryngeal granuloma, contact ulcers, and, as seen in this case, laryngeal webs. Chronic exposure of the laryngeal tissues to acidic gastric contents can incite a cascade of inflammatory responses, leading to structural changes, including thickening and pachyderma of the laryngeal tissue [5–9]. Figure 3 shows the possible pathophysiology for development of web secondary to reflux disease. The patient’s symptoms of recurrent globus, belching, and chest discomfort were likely due to reflux of gastric contents into the larynx and pharynx [3, 5–9]. Interestingly, the patient did not have typical symptoms of airway obstruction such as dyspnea, stridor, or voice changes, despite the presence of a posterior glottic web on laryngoscopy and CT imaging. This highlights the variability in clinical presentation of laryngeal webs, which can range from asymptomatic to severe airway compromise [1–3, 5–9].

The management of laryngeal complications, particularly LPRD secondary to GERD, primarily focuses on treating the underlying reflux disease. In this case, the patient was started on PPIs and a quadruple *H. pylori* eradication regimen, which represent the standard approach to reducing gastric acid secretion and minimizing reflux episodes [5–9]. The marked symptomatic improvement observed highlights the critical role of effective acid suppression in managing reflux-related laryngeal conditions. These results are consistent with findings from previous studies, which report a high rate of symptom resolution and normalization of laryngoscopic findings following appropriate anti-reflux therapy [3, 6–10].

Literature indicates that symptomatic laryngeal webs are typically managed surgically, with laser excision being a common approach [1, 2, 4, 10]. Most reported cases of posterior laryngeal webs have required surgical intervention due to respiratory distress or dysphonia. However, our case highlights a milder presentation successfully managed with conservative therapy. As the web was suspected to be secondary to chronic epithelial inflammation from LPRD and GERD, and given the absence of respiratory compromise, we opted for anti-reflux therapy alone, which led to significant symptom improvement. This suggests that in select cases where the web is incidentally detected and symptoms are mild or attributable to underlying reflux, a non-surgical approach may be appropriate. In contrast, refractory or symptomatic cases continue to warrant surgical intervention.

We acknowledge the lack of follow-up laryngoscopy, leaving uncertainty about spontaneous resolution despite symptomatic improvement and minimal airway compromise in our case.

This case illustrates the potential of GERD to cause laryngeal complications, including posterior laryngeal webs. The patient’s successful symptom resolution through reflux-targeted therapy underscores the importance of recognizing and treating GERD in patients with laryngeal complaints. Clinicians should maintain a high index of suspicion for reflux-related laryngeal involvement, as early treatment may prevent complications and improve outcomes. Future studies should explore the mechanisms linking GERD to such rare laryngeal findings and evaluate long-term outcomes of conservative versus surgical management. Key clinical insights are summarized in Table 2.

**Table 2:** Key learning points.

Key learning points
Chronic laryngeal inflammation from LPRD/GERD may be a potential contributing factor in the development of posterior webs, even without prior trauma or intubation. Conservative management with anti-reflux therapy can be effective in select asymptomatic or mildly symptomatic cases. Surgical intervention should be considered in patients with significant airway compromise or persistent voice-related symptoms. Incidental laryngoscopic findings should always be interpreted in the context of clinical symptoms and history.
